# Learn to Train: Improving Training Data for a Neural Network to Detect Pecking Injuries in Turkeys

**DOI:** 10.3390/ani11092655

**Published:** 2021-09-09

**Authors:** Nina Volkmann, Johannes Brünger, Jenny Stracke, Claudius Zelenka, Reinhard Koch, Nicole Kemper, Birgit Spindler

**Affiliations:** 1 Institute for Animal Hygiene, Animal Welfare and Animal Behavior, University of Veterinary Medicine Hannover, Foundation, 30173 Hannover, Germany; jenny.stracke@tiho-hannover.de (J.S.); nicole.kemper@tiho-hannover.de (N.K.); birgit.spindler@tiho-hannover.de (B.S.); 2 Department of Computer Science, Faculty of Engineering, Christian-Albrechts-University, 24118 Kiel, Germany; jobr@informatik.uni-kiel.de (J.B.); cze@informatik.uni-kiel.de (C.Z.); rk@informatik.uni-kiel.de (R.K.)

**Keywords:** artificial intelligence, animal welfare, injury detection, semantic segmentation

## Abstract

**Simple Summary:**

Injurious pecking against conspecifics in turkey husbandry is a widespread, serious problem for animal welfare. Evidence suggests that bloody injuries act as a trigger mechanism to induce pecking. Thus, continuous monitoring of the herd should be ensured to allow timely intervention in this type of behavior. The aim of the present study was therefore to develop a camera-based warning system using a neural network to detect injuries in the flock. The data for the network were provided by images on which human observers marked existing pecking injuries. Then, a network was trained with these human-labeled images in order to learn to detect pecking injuries on other unknown images from the same domain. As the initial agreement on the injuries detected by human observers and the trained network was unacceptable, various work steps were initiated to improve the data that were used to train the network. Finally, the aim of this process was for the network to achieve at least a similar ability to mark injuries in the images as a trained human observer.

**Abstract:**

This study aimed to develop a camera-based system using artificial intelligence for automated detection of pecking injuries in turkeys. Videos were recorded and split into individual images for further processing. Using specifically developed software, the injuries visible on these images were marked by humans, and a neural network was trained with these annotations. Due to unacceptable agreement between the annotations of humans and the network, several work steps were initiated to improve the training data. First, a costly work step was used to create high-quality annotations (HQA) for which multiple observers evaluated already annotated injuries. Therefore, each labeled detection had to be validated by three observers before it was saved as “finished”, and for each image, all detections had to be verified three times. Then, a network was trained with these HQA to assist observers in annotating more data. Finally, the benefit of the work step generating HQA was tested, and it was shown that the value of the agreement between the annotations of humans and the network could be doubled. Although the system is not yet capable of ensuring adequate detection of pecking injuries, the study demonstrated the importance of such validation steps in order to obtain good training data.

## 1. Introduction

Pecking behavior toward conspecifics is a serious animal welfare problem in turkey husbandry. Turkeys that are pecked by their conspecifics can suffer serious injuries, even leading to the death of the victims or the need for emergency euthanasia [1]. As bloody injuries act as a trigger mechanism to induce further pecking [2], early detection of such an occurrence and intervention by selecting and separating the injured animals can avoid further and persistent wounding. In addition, early initiation of management measures (such as additional environmental enrichment [3] and short-term reduction in light intensity [4]) is crucial in order to prevent the death of individual animals caused by massive injuries and to alleviate the situation within the affected herds [5]. The topic of preventing such serious pecking injuries is also gaining relevance in order to stop the use of the prophylactic, but painful, method of beak trimming [6], which should be banned in Germany in the future [7]. With the implementation of this ban, the problem of pecking injuries in the turkey flock will probably increase as it is more difficult to keep turkeys with intact beaks in the current conventional housing conditions [3,8].

Since injurious pecking behavior can spread quickly in the flock, many animals can be affected within a very short time; thus, timely intervention is highly relevant [5]. However, such timely intervention by the turkey farmer is heavily dependent on the frequency and time of walk-through inspections. Finding injured animals is complicated due to the fact that in large groups of several thousand animals, as is usual under commercial production conditions, purely visual monitoring of the flock is usually difficult and constant attention is practically impossible [9]. Therefore, applications of new technologies as support systems to the turkey farmer have been investigated in poultry husbandry to monitor broiler activity [10,11], broiler health [12,13] and turkey and broiler behavior [14,15].

In order to support the turkey farmer in monitoring the flock with regard to animal welfare-related issues such as the occurrence of injurious pecking, the main contributions of this study are the detection of occurring injuries using a neural network as well as improving the training data through costly annotation work steps in order to enhance its performance.

The remainder of this paper is organized as follows. Section 2 presents a survey of related work. Animals, materials and methods are described in Section 3 followed by the results in Section 4. The findings are discussed in Section 5, while conclusions are drawn in Section 6.

## 2. Related Work

This section discusses related work in the field of monitoring techniques using computer vision systems, managing the pecking behavior of turkeys as well as applying the annotation method of semantic segmentation.

One option for implementing the visual monitoring of a turkey flock is the use of computer vision systems, which can provide non-intrusive, non-invasive, and objective assessments [16]. Hitherto, previous studies have also developed computer vision systems to observe animal welfare-related issues, such as weight [17], temperature [18], thermal stress [19], and/or lameness [20,21]. These studies only include research concerning broiler chickens, whereas, to the best of our knowledge, no computer vision systems for monitoring animal welfare in turkey husbandry exist.

Pecking behavior and injurious pecking in turkeys in terms of animal welfare have been examined in previous studies [22,23]. Gonzales et al. recently developed an automatic pecking activity detection system based on audio data that could serve as a warning system for pecking injuries and possible cannibalism [14]. These researchers investigated how pecking activity changed over the fattening period and showed satisfactory accuracy in monitoring object-pecking activity. However, they could not provide an answer to the question of whether the onset of cannibalism/injurious pecking could be detected by changes in object-pecking frequencies or not. Bartels et al. [24] documented the behavior of male turkeys and the circumstances surrounding pecking injuries. They concluded that injurious pecking events in male turkeys resulted from agonistic interactions in the context of ranking fights, and even seriously injured animals were continuously maltreated by larger groups of ten birds. These findings increase the need to develop a system that is capable of detecting such injurious pecking behavior in the flock in a timely way.

Image segmentation using the annotation method of semantic segmentation was used previously in the field of livestock research. Xu et al. described a machine-learning-based quadcopter vision system for the classification and counting of sheep and cattle, where each animal on the processed images was labeled with semantic segmentation [25]. A combined method of instance and semantic segmentation was investigated to trace the contours of pigs by Brünger et al. [26]. Their method provided a pixel-accurate segmentation of the pig and achieved very good detection rates (F1-score: 95%) when tested on the used data set. In the area of poultry science, semantic segmentation was previously used in a study that presented a framework for automatic visual inspection of poultry viscera [27]. The authors obtained a ground-truth by manually annotating pixels belonging to the classes of interest, as they needed this to train and validate their supervised learning algorithms [27].

The use of artificial intelligence (AI)-enabled technology in turkey husbandry using automated visual detection of pecking injuries can prevent animal suffering and economic losses due to production concerns [28]. In order to support turkey farmer in monitoring the animals, the developed system should at least achieve similar results when assessing the flock as a human observer does. Therefore, as described in the introduction, the aim of this study was to develop a monitoring system for pecking injuries in the flock and adjust the detection performance of the network to the level and accuracy of human observation through the use of various work steps. Thus, since a network can only work as well as its “trainer” [29], the particular focus of this study was on improving training data.

## 3. Animals, Materials, and Methods

In this section, general information regarding the image acquisition and processing is given. The software and method for annotation are introduced, and the individual steps carried out on and with this software in order to improve the data for training a neural network to detect pecking injuries in turkeys are described.

### 3.1. Data Collection

Observations were conducted on a German research farm with female turkeys of the strain, British United Turkeys 6 (B.U.T. 6) (Aviagen Group, Newbridge, UK) with untrimmed beaks. The birds (*n* = 2170) were housed for rearing and fattening in a Louisiana type barn (29.2 × 15.9 m) with natural and forced ventilation systems. The lighting regime in the barn was implemented by means of artificial light sources (at least 20 lux) in addition to natural light and blinds for darkening. In the barn, three top view video-cameras (AXIS M1125-E IP-camera, Axis Communications AB, Lund, Sweden) were installed approximately 3 m above the ground. Video recordings with a resolution of 1080 P and set to record 25 frames per second were started one week after the chicks moved in and the chick guards were opened, thus allowing the animals to move around freely in the barn. The turkey hens were recorded two days per week (Tuesday and Thursday: 9:00–16:00) during their entire fattening period of 16 weeks, and two periods were observed. For further processing, the video recordings were divided into individual frames (6300 images per video) and were analyzed using annotation software.

### 3.2. Annotation Assessment

The detection of the injuries was performed with a segmentation network, which generates a semantic segmentation for each pixel in the image and assigns it to the class “injured” or “not injured”. For the generation of the training data through the detection of injuries by the human observers, a web tool was developed that made pixel-accurate marking of the visible injuries possible. This enabled the observers to work in a decentralized manner while keeping the data centralized. The images to be annotated were presented to the observers in a zoom/pan view (client-side Javascript) while the marked pixels were stored in an octree data structure. A separate drawing layer was created for each injured animal so that the number of injured animals in the image could be recorded simultaneously. The observers used a freehand drawing tool to mark each injury with a colored layer and applied different colors for individual turkey hens. The chosen method of annotation, that is, labeling each pixel of an image as being part of a potential injury is called semantic segmentation. When an annotation was saved, the data was transferred to the server and stored in a database. The labeled images needed for the training of the segmentation network could then be easily generated using this database by transferring the pixel positions stored in the octree back into an image.

Formally, the neural network was trained with the annotated data to minimize the following loss function:

L(gt, pr) = −gt α(1 − pr)^γ^ log(pr) − (1 − gt) α pr^γ^ log(1 − pr)

where gt is the annotated “ground-truth” and pr is the network’s prediction. This Focal Loss [30] is an extension of the classical Cross Entropy Loss, which takes into account the unequal distribution of the pixels with the classes “injured” or “not injured” by using the weighting factors α (set to 0.25) and γ (set to 2.0).

**Figure 1:**
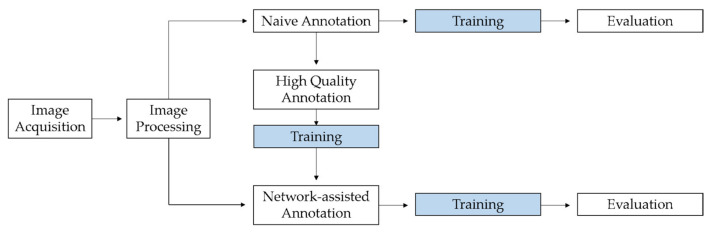
Flow chart showing the various work steps in the study, starting with image acquisition and processing. Processed images were used for naive annotation and network-assisted annotation. In addition, the intermediate step is shown in which high-quality annotations were generated, which were used for additional training of the network. In order to determine the benefits of the intermediate step, the results of the network after the training with naive annotations and with network-assisted annotations were compared.

After two months of naive annotation (see Figure 1), intra- and inter-observer agreement in the pixel-exact labeling annotation of injuries was tested. Thus, in the first test, which measured inter-observer agreement, 50 randomly selected images from the pool of extracts of the video-recordings were examined by the observers and for intra-observer reliability, one observer rated these images twice with a time interval of five days. A second test of inter-observer agreement was performed after three additional months of processing. At this point, 132 images (20 images from 12 different recording days) were randomly selected, rated by the human observers, and tested for pixel-exact agreement. To examine the performance of the assessment and the segmentation, the intersection over union (IoU) was calculated whereby IoU serves as a standard performance measure for segmentation [31] and evaluates the deviation between ground truth and predicted areas [32].

In this first step of the annotation procedure (naive annotation), two human observers processed the images from which a random part was then used to train and evaluate a neural network for semantic segmentation (a U-Net based on an Efficient-Net backbone [33,34]) by using 80% of the images for training and 20% for validation. Training was performed with an “early stopping” feature to avoid overfitting because the training is stopped automatically when a chosen metric stopped improving [35]. The pixel-exact agreement between human observers and network was then calculated for the detected and annotated injuries.

The next step was to generate high-quality annotations (see Figure 1). Therefore, the software for annotation was modified and the images were partitioned into small pieces (600 × 600 pixel) on which one labeled potential injury was shown. Then, the naive annotations were evaluated by presenting them to the human observers for a second time (*n* = 3). In this modified software, a binary system was created as the observer had to decide whether the annotation shown was actually an injury or not (injury vs. no injury) while being blinded as to whether it was originally annotated by the network or by a human. According to the work-flow, an image piece should be rated by three observers before it was saved as “finished” with the respective opinions. Therefore, an image piece for which an evaluation was already available was presented to a second observer at the earliest opportunity, and so on, until three opinions existed. Furthermore, the observers had to determine the location of the selected injury and could choose between injuries to the plumage, the head, or the snood. While performing the evaluations, it was also possible to reject single image pieces due to poor quality or incorrect annotation. In this case, all the image pieces from the entire original image were placed on a blacklist.

In the additional validation process, the high-quality annotations were used as further training data for the network. Therefore, the detections on the images were colored according to the agreement, for example, by coloring the agreements green and the disagreements red. Random sections were then chosen from these pictures. If there was disagreement on the pixels in such a section, for example, the red-colored, this section was rejected, and a new one was selected. By operating in such a manner, the data set used to train the network included image sections with and without injuries but never contained sections of disagreement. Subsequently, the dataset was split randomly, and the network was trained again.

The software for the naive annotations from the first step was expanded to include a further function, a human-in-the-loop approach was implemented, and on request, the network trained with high-quality annotation could generate detections on unknown images. The labeled detections were then presented to the human observers (*n* = 2) for review. Afterwards, the images were edited by the human observers who could add, delete, or revise networks markings. The neuronal network was then trained with these network-assisted annotations (see Figure 1).

Finally, whether the agreement between pixel-exact annotations of the network and human observers could actually be improved through use of the intermediate step with the high-quality annotations was tested by comparing the results after the training with naive annotations and with network-assisted annotations.

## 4. Results

### 4.1. Naive Annotations

Using the first-developed software, more than 19,500 images were annotated from which 3400 images were randomly selected to be used as training data. This selection was done to allow a fair comparison with the same-sized training data set in the network-assisted annotation as described in Section 4.3. In the tests of intra- and inter-reliability, the pixel-exact agreement between the human observers for annotating all pecking injuries on the shown images ranged from 0.25 to 0.43 IoU at maximum (Table 1). These values were regarded as unacceptable based on the classification values proposed by Landis and Koch: (1) <0.00 = poor, (2) 0.00–0.20 = slight, (3) 0.21–0.40 = fair, (4) 0.41–0.60 = moderate, (5) 0.61–0.8 = substantial, and (6) 0.81–1.00 = almost perfect [36]. 

Even the intra-observer reliability, with a value of 0.56 IoU, achieved only a slightly moderate agreement (Table 1). Of course, IoU is a rather strict metric and compares the observers’ annotations as pixel-exact values, a process that makes the inaccuracies between different observers more apparent.

A slightly different metric was used to evaluate the agreement between human observers and the trained network, which did not evaluate the exact pixel-exact segmentation as strictly, but rather the robust detection of the injuries. Based on classical detection methods, the individual annotated or detected injuries were outlined with a bounding box. The individual bounding boxes could then be classified into successful detections (true positives), false detections (false positives), and missed detections (false negatives) using the IoU and a threshold of 0.5. From this process, the metrics precision and recall, and the combined metric F1-score could be derived. The results of the comparison between the human observers and the network are listed in Table 2.

### 4.2. High-Quality Annotations

**Figure 2:**
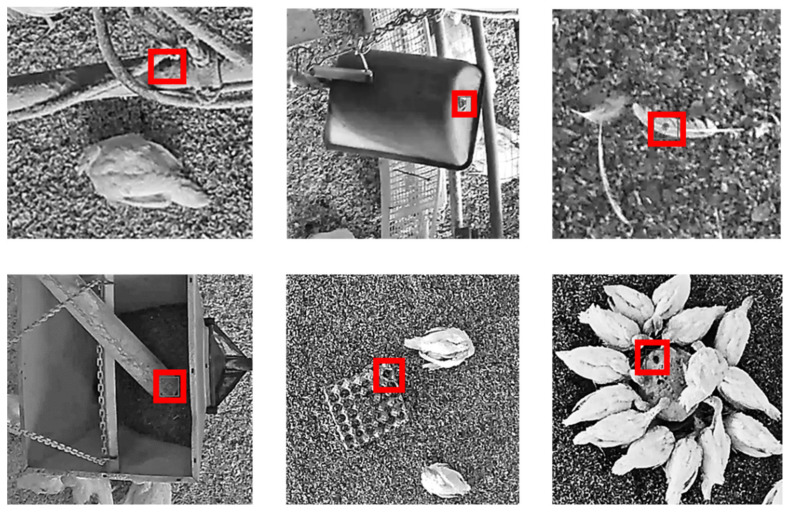
Example images of annotations of the network (on stable equipment and pecking objects in the litter), which were clearly assessed as “no injury” by human observers creating high-quality annotations. The originally annotated areas on the sample images (which were shown in black and white for better visibility) were framed with red bounding boxes for easier identification.

**Figure 3:**
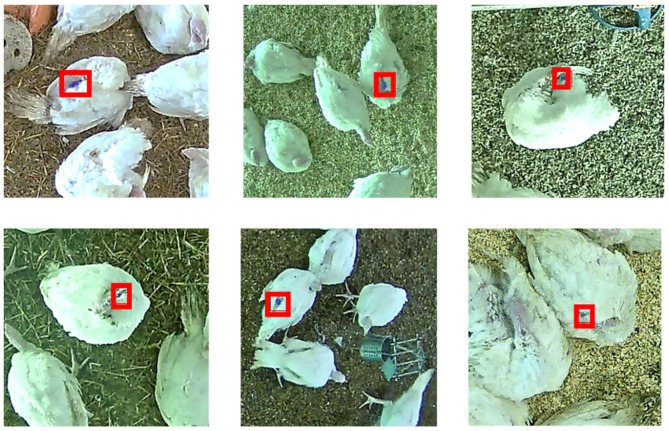
Example images for annotations of the network in which the human observers creating high-quality annotations did not agree since a clear assessment was difficult due to shadows, lighting, view of camera, and/or the animals’ posture. The originally labeled annotations on the sample images are blue-colored and framed with red bounding boxes for easier identification.

In regard to the annotation from the first step, a total of 24,173 opinions were given by the observers and stored in the modified software for the assessment of high-quality annotations. This total number resulted in 6895 annotations for which there was the requested three opinions of the human observers. Of these, 865 annotations (12.6%) were rated as “no injury” (example images are shown Figure 2) and 6030 as “injury” (87.4%). Of the injuries, (*n* = 5339) were assigned to the feathered body region (plumage), 638 to the head, and 53 to the snood. All three observers agreed on 5621 annotations (81.5%), but this was not the case in 1274 cases, where they disagreed on 18.5% of the annotations (example images are shown in Figure 3). In this context, the observers agreed on 81.5% of the injuries to the plumage, on 90.8% of those to the head, and on 98.2% of the injuries to the snood. Of 6895 annotations, a total of 1363 images were evaluated and shown to be high-quality annotations, whereby an image can contain several annotations.

### 4.3. Network-Assisted Annotations

**Figure 4:**
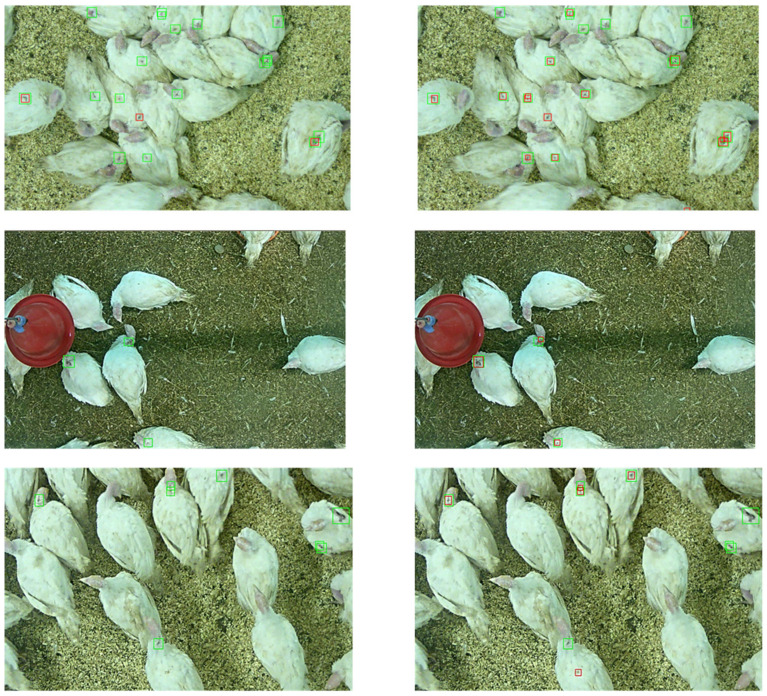
Example images that were annotated by the neural network. On the left side, the network was trained with 3400 images labeled with naive annotations of human observers; on the right side, it was trained with 3400 images labeled by network-assisted annotations obtained from human observers. The human annotations are framed with green bounding boxes and those of the network are framed with red bounding boxes.

A total of 3400 images were edited as network-assisted annotations. After further training, the network yielded an agreement with the human observers of F1-score = 0.14, a recall of 0.19, and precision of 0.11 (Table 2). Thus, the agreement between humans and the network trained with the naive annotations doubled in respect to humans and the network trained with network-assisted annotations (F1 = 0.07 to F1 = 0.14) (Figure 4).

## 5. Discussion

The aim of the present study was to develop an automated system through AI that is capable of monitoring ongoing pecking injuries in a turkey flock. Parameters, such as temperature, are currently measured constantly in conventional turkey husbandry, but a monitoring system based on animal behavior for the occurrence of injurious pecking has not been reported yet [37].

### 5.1. Quality of Raw Data

One challenge in annotating the images recorded in the turkey flock was the quality of the raw data. Although the cameras had a high resolution, and therefore a high number of pixels in an image, a good sampling rate with 25 frames per second, and suitable installation (meaning an appropriate distance between the camera and surface of interest [32]), several issues were identified that made it difficult to detect injuries.

The quality of the raw data could have been poor due to the physical action of the birds and/or whirling dust. Furthermore, the detection of injuries was complicated by the fact that the lighting conditions in the barn were not consistent. For instance, in summer, when direct sunshine intensifies, the barn is darkened by means of green blinds, and thus bloody red wounds were discolored. This type of darkening is also commonly used as a management measure when injurious pecking increases in the flock [4]. The darkening and green light in the barn made it more difficult for human observers to evaluate the annotations. However, in this study, the extent to which the detection of the network was influenced by these circumstance was not determined.

The results of the assessment for obtaining high-quality annotations showed that the human observers were particularly not in agreement regarding injuries to the plumage. These plumage injuries were difficult to assess correctly when the turkeys were not in a standing position with its wings back, but instead, for instance, cleaning itself, resting on the floor, or sleeping with its head placed on its back (see Figure 3). In these situations, shadows were cast, which made the assessment difficult compared to detecting a potential injury on the body of a white-feathered turkey. Comparable results were reported for the detection of tail lesions in pigs where blurry images or discoloration complicated the observations and image assessments [29]. More generally, classifying images into discrete categories is sometimes quite unnatural for humans, which is why there is also research on classification based on soft labels [38].

Furthermore, high-stock density, which leads to overlapping and occlusion, has been mentioned as a reason for the complicated annotation of images showing poultry [32]. The recordings in the flock (not under laboratory conditions) indicated that environmental impacts, such as drinkers, feeders, and/or feathers on the ground could negatively influence the performance, especially of the network detection (see Figure 2).

### 5.2. Annotation Method

The prerequisite for training a network to detect and annotate injuries is a large number of images, which are first labeled by humans. Thus, the network can use these annotated images as a template. The different methods for annotating images depend on the visual template and the “object” that has to be labeled. Various tools are available for the annotation of images, and these tools process the information displayed on the image and make it “understandable” for the AI. One way to make objects recognizable on an image is to use bounding boxes that show a drawn frame around the object. For non-rectangular objects, bounding boxes have the disadvantage of large overlap since they also contain a large area that is of no interest (for instance the background) or even parts of other animals [26]. Another method of annotation is marking with ellipses, which can be easily drawn and were used in previous studies, for example, to reproduce the bodies of pigs [39,40]. The highest level of accuracy is obtained by pixel-exact labeling of the object in color on the screen using semantic segmentation, indicating that every single marked pixel is assigned to a class [41]. In the present study, these classes were defined as injuries of the individual animals on the images. Although this semantic segmentation is very costly and a pixel-exact match is very difficult to achieve, we chose it as the annotation method for this study because, in this way, the shape of individual injuries can be marked most correctly. The aim was to record both the extent of the injuries and the number that were marked per picture. Previous studies used semantic segmentation, for instance, in algae detection [42], sow nursing behavior [43], detection of the respiratory rates [44], and monitoring the feeding behavior of dairy cows [45]. Of course, this exact annotation method can also be a source of error since with pixel-exact labeling, the smallest deviations in the human performance increase. For this reason, agreement values in the case of pixel-exact annotation cannot be compared with results from other studies using bounding boxes or ellipses fitting.

### 5.3. Improving Training Data

With the work steps and software development, the training data for the neural network should have been improved in a manner similar to Brünger et al. [29] who stated “in supervised learning like the one presented here, neural networks can only be as good as the data they are trained with”. In the present study, the network learned from the manually annotated data obtained from human observers. To support the turkey farmer in monitoring a flock of several thousand turkeys, the network should detect pecking injuries as accurately and securely as possible.

In performing the naive annotations, the agreement between the network and the humans reached a F1-score of 0.07 with a precision of 0.21 and a recall of 0.04. As the precision was measured as the ratio of true positive detection over all detections, a low value of 0.21 indicates a high rate of false positive detections. Even worse, a recall of 0.04 indicates an even higher number of false negative detections, since the recall measures the proportion of annotated detections that were detected correctly. Such numbers strongly indicate the need for improvement in the training data.

However, since the human observers also had considerable problems in uniformly, continuously, and reliably detecting potential injuries (see Figure 3), a correct human assessment should next be achieved by including additional and labor-intensive intermediate work steps. In this process, the annotations of three trained observers should be compared and evaluated in order to generate high-quality annotations as training data for the network. Therefore, during the work process, the original naive annotations were verified by three human observers by requesting three opinions of the labeled annotation (injury/no injury) before an image section was considered “finished”. Such a verification process was done for all the detections that were present on one image. Due to the limitations of staff and the workload, significantly fewer images were annotated in this work step for generating high-quality annotations than in the other ones. Further processing of the 1363 images also ensured that the network saw sections of the picture with and without injuries but never pictures that contained unsafe areas and only sections of the picture on which the three observers agreed.

With this costly working step, much effort was put into training a network in order to deliver only high-quality annotations to the network and to subsequently reduce the workload for the human observers in the following annotation process due to pre-marking of the network generating the network-assisted annotations.

After training with the network-assisted annotations, the agreement between the network and the humans reached a F1-score of 0.14 with a precision of 0.11 and a recall of 0.19. Compared to the network that was trained with the naive annotations, the precision decreased, indicating slightly more false positives. More importantly, the recall increased significantly to a value of 0.19. This finding indicates that the network yielded slightly more false positive detections but no longer missed any injuries. As the long-term goal of the monitoring system to be developed is to detect injuries reliably and early enough to take the necessary steps to stop these undesired behaviors, these results can be considered as a good starting point.

An F1-score of 0.14 in the annotation is certainly not an outstanding value. Since the agreement was calculated and averaged over all detections, it is feasible that the network also labeled all annotations on an image in a manner similar to the human observers but labeled the annotation to a lesser degree than did the humans. Such differences in size naturally lead to differences in the number of labeled pixels, which leads to the reduced probability of a match. However, the intensive work on high-quality annotations resulted in a doubling of the F1-score.

### 5.4. Choice of Neural Network Architecture

As described in Section 3.1, the automatic detections were generated using a segmentation network with a U-Net architecture [33]. The basic design of the network follows the original work in [33], but uses a more up-to-date classifier as the encoder backbone. During the development of the training pipeline, other network architectures such as FPN [46] and PSP [47] were also tested, but these gave worse results. However, as discussed in the other sections of this chapter, the ultimate choice of network architecture is not critical until the generation of high-quality training data and the definition of precise injury assessment standards are finalized.

### 5.5. Limitations and Future Research

The present research has several limitations. First, although the images used to train the network originated from different places with different environmental equipment, they were always obtained from the same barn. In addition, potential injuries were detected in turkeys of the B.U.T. 6 breed, all of which have white plumage. Therefore, it is not known whether such a system can also be used for animals with other plumage colors. Only observing white birds was also mentioned as a limitation in a previous study that aimed to provide a methodology to identify bird behavior using combined techniques of image processing and computer vision [15]. Since the common breed for fattening has a white plumage, and is thus particularly suitable for monitoring in a flock, the color of the turkeys’ plumage may not be the most severe limitation of the study.

Certainly, the biggest issue in providing good training data was the unreliable detection and annotation of injuries that then led to disagreement between the human observers. This problem occurred primarily in the case of plumage injuries in which reliable detection was made difficult by cast shadows, turkeys’ posture, and/or overlapping of the individual animals (see Figure 3). When generating high-quality annotations, the human observers were in less agreement on plumage injuries compared to injuries to the head or snood. Without a specified “gold standard” for annotation or a verification of the correctness of a detection [48], the evaluations of the images were based solely on the experience of the observers. It might be possible to secure results by one person evaluating images from the flock while another observer verifies them in the barn by means of live observation. Another alternative is for several trained observers to annotate the same image, and afterwards a calculated mean value for the annotated pixels could be used for further processing. Thus, Muñoz et al. [49] improved training data by using averaged annotations from several trained panelists who were evaluating marbling. In their study, Brünger et al. [29] performed five inter-observer tests before a satisfactory agreement was achieved, and only then was the image assessment for tail lesions started. In the present study, the observer reliability tests were carried out in the course of the assessment, and the annotations were not only carried out after a certain agreement was reached. However, the procedures used in the annotation assessment are not comparable since Brünger et al. [29] used a score for rating tail lesions, and in this scenario, the pixel-exact agreement of the annotations was tested.

With regard to the neural network, there were several faulty detections concerning stable equipment, the litter, and/or the feathers lying around which were annotated (see Figure 2). These wrong annotations could be avoided if the system, for instance, was able to differentiate between turkeys and background. Thus, the network could learn that it can only be a “true” injury if the labeled pixels are placed on an animal; this would certainly help improve the detection accuracy. Thus, this aspect could be taken into account in future research.

Currently, to our knowledge, no technology is available to actually measure animal welfare in turkey farms; however, an increase in scientific research and publications focused on animal health and welfare has been reported [50]. If the system to be developed for the detection of injuries should function reliably in the future, then for practical implications it could be used as a supplement to recording a welfare assessment protocol, for example [51]. Furthermore, the system would provide registered data that can be used to set individual thresholds and/or to make comparisons between the health status of the turkeys within or between farms. However, in general, the presented system still needs to be improved in order to achieve an accuracy level that makes it feasible to use it as a warning system in practice.

## 6. Conclusions

The primary aim of this study was to develop an image-based automated system using a neural network to detect pecking injuries in a turkey flock. Based on the reliable and uniform pixel-exact annotation of such injuries on images of turkey hens, which was used to train the network, various additional work steps were carried out to improve network training data. This process was achieved in the present study with a lot of annotation work, even though no outstanding results could be achieved. However, the different work steps involved can be viewed as meaningful even if the system itself still needs further improvement. The study showed that such validation steps are important for good training data and furthermore, it is desirable to develop such a system in order to provide timely monitoring of the animals. Nevertheless, the crucial point remains that such a warning system can only be used to draw attention to already existing pecking injuries in order to separate the injured animals or to offer the animals more environmental enrichment. Unfortunately, such a system does not offer a method that prevents such pecking behavior from the outset.

## Figures and Tables

**Table 1:** Pixel-exact agreement resulting from the first test (1) of inter-observer agreement between the three observers (OBS1–3) and intra-observer-reliability (OBS1 vs. OBS1a) and the second test (2) of inter-observer agreement between the three observers (OBS1–3).

Comparison	IoU ^1^ (1)	IoU ^1^ (2)
OBS1 vs. OBS2	0.27	0.30
OBS1 vs. OBS3	0.43	0.36
OBS2 vs. OBS3	0.25	0.29
OBS1 vs. OBS1a	0.56	

^1^ IoU = intersection over union.

**Table 2:** Results of the agreement between human observers and network after training with the naive annotations (NA) and the network-assisted annotations data (NAA). The intersection over union (IoU) is averaged over the individual detections and the values for F1-score, precision, and recall are listed.

	NA	NAA
F1	0.07	0.14
PRECESION	0.21	0.11
RECALL	0.04	0.19
